# Heat Shock Protein A6 Is Especially Involved in Enterovirus 71 Infection

**DOI:** 10.3389/fmicb.2022.865644

**Published:** 2022-03-04

**Authors:** Jiaoyan Jia, Ge Liu, Jianfeng Zhong, Ran Yan, Xun Song, Kai Zheng, Zhe Ren, Zhendan He, Qinchang Zhu

**Affiliations:** ^1^College of Pharmacy, Shenzhen Technology University, Shenzhen, China; ^2^School of Pharmaceutical Sciences, Shenzhen University, Shenzhen, China; ^3^Institute of Biomedicine, College of Life Science and Technology, Jinan University, Guangzhou, China

**Keywords:** HFMD, EV71, heat shock proteins, HSPA6, host factor, specificity

## Abstract

Hand foot and mouth disease (HFMD) caused by Enterovirus 71 (EV71) infection is still a major infectious disease threatening children’s life and health in the absence of effective antiviral drugs due to its high prevalence and neurovirulence. A study of EV71-specific host response might shed some light on the reason behind its unique epidemiologic features and help to find means to conquer EV71 infection. We reported that host heat shock protein A6 (HSPA6) was induced by EV71 infection and involved infection in both Rhabdomyosarcoma (RD) cells and neurogliocytes. Most importantly, we found that EV71 did not induce the expression of other heat shock proteins HSPA1, HSPA8, and HSPB1 under the same conditions, and other HFMD-associated viruses including CVA16, CVA6, CVA10, and CVB1-3 did not induce the upregulation of HSPA6. In addition, EV71 infection enhanced the cytoplasmic aggregation of HSPA6 and its colocalization with viral capsid protein VP1. These findings suggest that HSPA6 is a potential EV71-specific host factor worthy of further study.

## Introduction

Enterovirus 71 (EV71) from the Picornaviridae family is one of the common causative pathogens for hand foot and mouth disease (HFMD), which is usually a self-limiting, mild childhood disease with typical clinical manifestations of fever, mouth sores, skin rash on hands, mouth, and/or feet (Wong et al., 2010). Nevertheless, it can cause neurological complications such as myocarditis, aseptic meningitis, encephalitis, neurogenic pulmonary edema, and even death in severe cases. HFMD has emerged as a major concern among pediatric infectious diseases during the past 20 years, particularly in the Asia-Pacific (Koh et al., 2016). Countries such as Taiwan, Singapore, Malaysia, Vietnam, and China have encountered outbreaks of HFMD epidemics and millions of infections since the late 1990s. Around 13 million HFMD cases were reported in China from 2008 to 2015, including 123,261 severe cases and more than 3,322 deaths (Yang et al., 2017).

There are more than 20 enteroviruses that can cause HFMD (Klein and Chong, 2015), but EV71 is the most clinically significant serotype, being associated with severe disease outcomes more frequently than other common causes such as coxsackievirus A16 (CVA16) and A6 (CVA6). It was reported that around 60% of clinical HFMD and more than 70% of severe HFMD were caused by EV71 infection (Huang et al., 2015; Wang et al., 2015). It is estimated that around 0.2–1% of children with EV71 infection develop neurological complications (Lee and Chang, 2010; Aw-Yong et al., 2019). The reason why EV71 is more prevalent and more likely to cause severe disease when compared with other enteroviruses is currently largely unknown.

Recombination and mutation were reported to occur extensively throughout the genome of EV71 and to be possible key factors affecting the virulence of EV71 (Mandary and Poh, 2018). New EV71 variant arose from the recombination of part of the EV71 genome with 5’-NTR of poliovirus may have acquired virulence determinants to give rise to clinical complications (Brown and Pallansch, 1995). Although several mutations such as E145G/Q and D164E in VP1, LysP930 in 2A, GP272, UP488, and AP700/UP700 in the 5’-NTR are highly conserved in the neuro-virulent strains across different genotypes of EV71 (Li et al., 2011), there is still no solid evidence to confirm their roles in virulence. Excessive secretion of cytokines including IL-1β, IL-6, IL-10, IL-17A, monocyte chemotactic protein 1 (MCP-1), and IFN-γ were reported to be linked to EV71 severity (Aw-Yong et al., 2019). The single nucleotide polymorphisms (SNPs) of type I IFN receptor 1 (IFNAR1) were found to be associated with the susceptibility and severity to EV71 infection (Zou et al., 2015). EV71-specific host responses may be related to the prevalence of virulence of EV71, but they have rarely been studied.

Heat shock protein A6 (HSPA6, also known as Hsp70B’) is a member of the highly conserved heat shock protein 70 kDa (Hsp70) family, which generally plays a wide range of roles in cellular functions in health and disease (Evans et al., 2010; Rosenzweig et al., 2019). Besides HSPA6, more than 13 Hsp70 isoforms have been identified in the human Hsp70 family, classified as inducible or constitutive and organ or tissue specificity (Radons, 2016; Ambrose and Chapman, 2021). Although there is a high sequence homology between the members, a considerable difference in function has been observed in the Hsp70 family (Daugaard et al., 2007; Hageman et al., 2011; Radons, 2016). For instance, HSPA1A (Hsp70-1) is one of the major cytoplasmic stress-inducible Hsp70s that has a preferential contribution to DNA repair and tumor immune response (Multhoff et al., 1995; Duan et al., 2014). HSPA8 (Hsc70) is constitutively expressed in both cytoplasm and nucleus and is associated with cellular housekeeping functions, while HSPA9 (Hsp70-9) is expressed in mitochondria and is not induced by heat stress and is correlated with muscle activity and mitochondrial respiration (Radons, 2016). Although sharing high sequence identity (81–85%) and partly overlapping cellular stress-response functions with HSPA1A, HSPA6 has unique induction characteristics, different substrates, and thus potential distinct functions (Daugaard et al., 2007; Hageman et al., 2011; Deane and Brown, 2017). HSPA6 is a strictly stress-inducible Hsp70 in higher mammals such as primates, swine, and goats but not rodents (Parsian et al., 2000; Ramirez et al., 2015), and has varied expression patterns between cell types (Noonan et al., 2007; Banerjee et al., 2014; Kuballa et al., 2015), suggesting it is newly evolved Hsp70. HSPA6 has been reported to protect human neuronal cells from cellular stress (Deane and Brown, 2018) and might be associated with earlier recurrence of human hepatocellular carcinoma (Yang et al., 2015). Compared to other widely studied Hsp70s like HSPA1A, very little is known about the functions of HSPA6.

Hsp70 family has been positively involved in viral infection, but most studies focus on general Hsp70 without paying much attention to particular isoforms (Kim and Oglesbee, 2012; Yang et al., 2020). Hsp70 functions at distinct steps of the viral cycle in a virus or isoform-specific manner (Phillips et al., 1991; Lahaye et al., 2012; Manzoor et al., 2014; Taguwa et al., 2015). For instance, HSPA1 and HSPA8 but not HSPA2, HSPA6, and HSPA14 were found to be recruited to zika virus-induced compartments and required for virus replication (Taguwa et al., 2019). HSPA1A, HSPA1B, and HSPA8 are required for dengue virus infection at steps of entry, replication, and virion production, while HSPA5 and HSPA9 are not (Taguwa et al., 2015). On the other side, HSPA5 was found to contribute to the entry and replication of the Japanese encephalitis virus (JEV), a member of the Flaviviridae family-like dengue virus (Nain et al., 2017). In addition, Hsp70s have been reported to participate in the infection of several enteroviruses. HSPA1A was reported to promote coxsackievirus B3 (CVB3) translation initiation and elongation through the Akt-mTORC1 pathway (Wang et al., 2017). Hsp70 (actually HSPA9) was reported to facilitate EV71 infection *in vitro* as a supplementary receptor (Xu et al., 2019). HSPA8 (Hsc70) was found to assist EV71 replication through upregulating the activity of viral the internal ribosome entry site (IRES; Dong et al., 2018). Another study also reported that HSPA8 and HSPA9 regulated all phases of the EV71 life cycle (Su et al., 2020). However, so far, there is no study addressing the specificity of Hsp70 in the infection of HFMD-associated viruses.

We reported that HSPA6 was induced by EV71 infection and involved infection in both RD cells and neurogliocytes. For the first time, we also show that EV71 did not induce the expression of HSPA1, HSPA8, and HSPB1 under the same conditions, and several other common HFMD-associated viruses did not induce the upregulation of HSPA6. In addition, EV71 infection enhanced the cytoplasmic aggregation of HSPA6 and its colocalization with viral capsid protein VP1. These findings suggest that HSPA6 is a potential EV71-specific host factor worthy of further study.

## Materials and Methods

### Cell and Viruses

Vero cells (African green monkey kidney cells, ATCC, CCL-81), RD cells (human muscle rhabdomyosarcoma; ATCC, CCL-136), and U251 cells (human brain glioma cells, BNCC,341988) were cultivated in Dulbecco’s modified Eagle’s medium (DMEM; Gibco) supplemented with 10% fetal bovine serum, 100 U/ml penicillin and 100 μg/ml streptomycin at 37°C with 5% CO_2_. EV71 C4/BrCr strains and Coxsackievirus A16 (CVA16) were kindly provided by Dr. Tao Peng, Guangzhou Medical University, China. Coxsackievirus B1, B2, B3, A6, and A10 (CVB1, CVB2, CVB3, CVA6, and CVA10) were obtained from ATCC. The viruses were propagated, titred in RD or Vero cells, and used at the indicated multiplicity of infection (MOI). EV71 C4 is a clinically isolated strain (Xu et al., 2017), mainly used EV71 in this study.

### Transcriptome Analysis and RT-PCR

RD cells were infected with the EV71 virus at a multiplicity of infection (MOI) of 0.2 for 8 h, and then the cells were collected and subjected to RNA extraction and transcriptomic sequencing analyses as described previously (Huang et al., 2020). The transcriptomes were sequenced on Illumina HiSeq2500 by Gene Denovo Biotechnology Co. (Guangzhou, China). Gene differential expression analysis was performed using DESeq2 software on EV71 infected (V) vs. uninfected groups (Ctrl). The genes/transcripts with the parameter of false discovery rate (FDR) below 0.05 and absolute fold change ≥2 were considered differentially expressed genes/transcripts.

For RT-PCR, RD or U251 cells were treated and/or infected as indicated, followed by RNA extraction. RD cells were infected with EV71 at an MOI of 0.2 for 8 h or 24 h, while U251 cells were infected with EV71 at an MOI of 1 for 24 h or 48 h. An active compound pendulectin (PDL, 10 μM) was used as antiviral control. According to the manufacturer’s protocol, total RNA was extracted using TaKaRa MiniBEST Universal RNA Extraction Kit (TaKaRa, Shiga, Japan). RNA samples were subjected to cDNA synthesis with PrimeScript RT Reagent Kit (TaKaRa, Shiga, Japan). PCR amplified and detection was performed with TB Green Premix Ex Taq II (TaKaRa, Shiga, Japan) in the QTOWER3G real-time PCR system (Jena, Germany). The relative gene expression was determined by using the 2^-ΔΔCT^ comparative method. GAPDH was used as the normalizing gene. Quantitative RT-PCR primer sequences were as follows: HSPA6 forward (5’-AGTACAAGGCTGAGGATGAGGC-3’) and reverse (5’-AAGGACTTCCCGACACTTGTCTT-3’); HSPA1A forward (5’-GACGCGAAGCGGCTGATT-3’) and reverse (5’-TCGGGGTAGAATGCCTTGG-3’); HSPB1 forward (5’-TACATCTCCCGGTGCTTCACG-3’) and reverse (5’-ATCTCGTTGGACTGCGTGGC-3’); HSPA8 forward (5’- ACTCCAAGCTATGTCGCCTTT-3’) and reverse (5’-TGGCATCAAAAACTGTGTTGGT-3’). EV71 positive-strand forward (5’-GCAGCCCAAAAGAACTTCAC-3’) and reverse (5’-ATTTCAGCAGCTTGGAGTGC-3’); EV71 negative-strand forward (5’-TGCGTGGTGATGGTGGAGTT-3’) and reverse (5’-GCCACAGCAGGCAAACAGAG-3’); EV-A71 VP1 forward (5’-CACACAGGTGAGCAGTCATCG-3’) and reverse (5’-GTCTCAATCATGCTCTCGTCACT-3’); CVA6 VP1 forward (5’-TAAGCACGTGAGAGCTTGGG-3’) and reverse (5’-CCGCCTTCATAATCCGTGGT-3’); CVA16 VP1 forward (5’-AGCCGTGCTGGTCTTGTTAG-3’) and reverse (5’-TGGGGGACTAGCTCTCCATT-3’); CVB2 VP1 forward (5’-ACCGTGGACGATTACAACTGG-3’) and reverse (5’-ATGCCTAAACTCGGACCAACC-3’). GAPDH forward (5’-GATTCCACCCATGGCAAATTCCA-3’) and reverse (5’-TGGTGATGGGATTTCCATTGATGA-3’).

### Western Blot Analysis

Cells were treated as indicated and Western blotted as previously described (Liu et al., 2015). Briefly, the cells were lysed and assessed using 4–20% sodium dodecyl sulfate–polyacrylamide gel electrophoresis (SurePAGE, Bis-Tris gels, Genscript, Nanjing, China). Proteins were transferred to polyvinylidene difluoride membranes (Millipore, Bedford, MA, United States) and probed with primary antibodies against HSPA6 (sc-374589, Santa Cruz Biotechnology), HSPA1A (PA534772, Invitrogen), HSPA8 (PA5-27337, Invitrogen), HSPB1 (PA1017, Invitrogen), EV71 VP1 (GTX132339, GeneTex), CVA6 VP1 (GTX132346, GeneTex), GAPDH (ab9485, Abcam) or α-Tubulin (ab179484, Abcam). Protein bands were visualized with Pierce™ ECL Western blotting substrate (Thermo Scientific, Rockford, IL, United States). Protein levels were determined by scanning the bands’ intensities and analyzed using Quantity One software (Bio-Rad, Hercules, CA, United States).

### RNA Interference (RNAi)-Directed Knockdown

RD cells in 12-well plate were cultured to 60–80% confluence and then transfected with siRNAs targeting HSPA6 gene or SCARB2 (Scavenger Receptor Class B Member 2) gene or negative control siRNA targeting no known genes (si-NC) using Lipofectamine™ RNAiMAX Transfection Reagent (Life Technologies, Carlsbad, United States) according to the manufacturer’s protocol. siRNAs were chemically synthesized by Guangzhou Ruibo Biotech Co., Ltd., China. Cells were infected with the virus 24 h after transfection. After 48 h of infection, the gene and protein expression levels were evaluated with RT-PCR and Western blotting, respectively. After 72 h of infection, the culture supernatants were collected, and the daughter virus production was analyzed with plaque reduction assay. At the same time, the cytopathic effect (CPE) caused by EV71 infection was measured by Alamar blue reagent as described previously (Zhu et al., 2011).

### Confocal Microscopy Analysis

RD cells grown on a glass-bottomed dish were infected with EV71 C4 strain (MOI = 0.2) for 8 h or 24 h and then were fixed with 4% paraformaldehyde and permeabilized with 0.1% Triton X-100. Then the cells were blocked with 5% BSA and then incubated with the indicated primary antibodies in 1% BSA at 4°C overnight. After three washes with PBS, the cells were incubated with a secondary antibody in 1% BSA for 1 h at room temperature in the dark and washed three times with PBS. The samples were then exposed to VP1 (GTX132339) / Alexa Fluor 488 or HSPA6 (sc-374,589) /Alexa Fluor 594 (Beyotime Biotechnology, Jiangsu, China) and observed under a Nikon A1 confocal microscope. The nuclei of the cells were stained with 4,6-diamidino-2-phenylindole (DAPI, Molecular Probes). Images and Z-stacks were acquired with NIS-Elements imaging software. Orthogonal views were generated in Imaris Software.

### Plaque Reduction Assay

RD cells were first transfected with siRNA against HSPA6 or SCARB2 or negative control siRNA (si-NC) for 24 h as described above. Then, the cells were infected with the EV71 C4 strain. After 48 h of infection, the culture supernatant was collected and subjected to plaque reduction assay as described previously (Zhu et al., 2011). The cells were finally stained with 1% crystal violet. The titer of the virus in the culture supernatant was calculated according to the number of forming plaques.

### Statistics Analysis

All the data presented herein are mean ± standard derivation values. Statistical comparisons were analyzed with a two-tailed Student’s t-test or one-way ANOVA followed by Dunnett’s post-hoc test using GraphPad Prism version 8.0.2 for Windows (GraphPad Software, California, United States). ^∗∗^Indicates *p* < 0.01 and ^∗^ indicates *p* < 0.05. *p* values <0.05 were considered statistically significant.

## Results

### EV71 Infection Specially Upregulates HSPA6 Gene Expression

**Figure 1:**
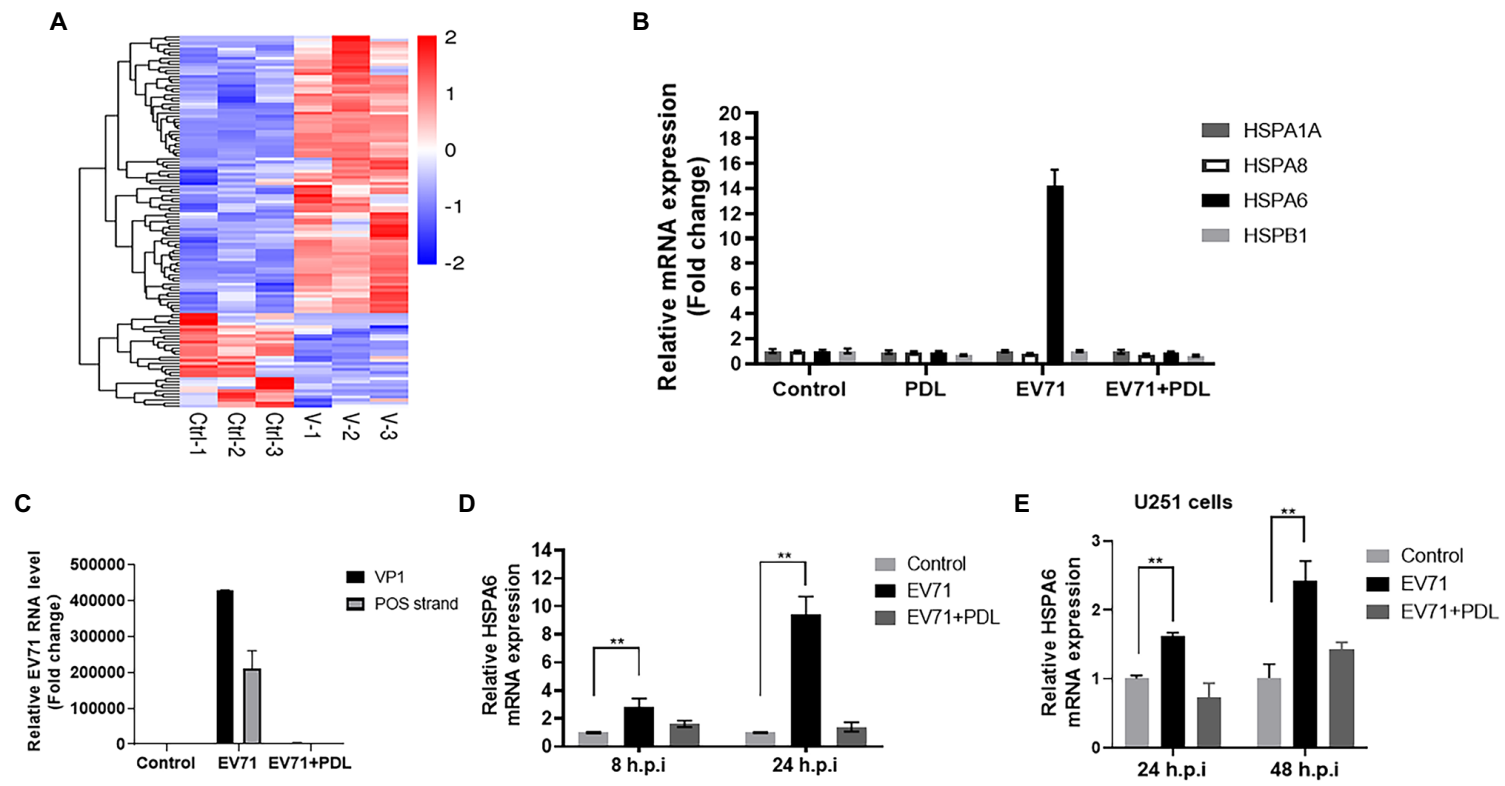
EV71 infection specially upregulates HSPA6 gene expression. **(A)** Heat map of RNA-Seq transcriptome analysis for genes changed in RD cells infected with EV71 for 8 h. **(B)** RT-PCR measurement of HSPA6 and HSPA1, HSPA8, and HSPB1 expression in RD cells infected with EV71 for 8 h. **(C)** RT-PCR measurement of EV71 VP1 gene and positive (POS) strand in RD cells infected with EV71 for 8 h. **(D)** RT-PCR measurement of HSPA6 expression in RD cells infected with EV71 for 8 h and 24 h. **(E)** RT-PCR measurement of HSPA6 expression in U251 cells infected with EV71 for 24 h and 48 h. Control presented cell control, and PDL presented treating the cells with 10 μM of pendulectin at the same time of EV71 infection (^**^p < 0.01).

Comparative transcriptome analysis was firstly performed and found that the expression of HSPA6 mRNA was upregulated in EV71 infected RD cells (Figure 1A), which could be inhibited by the treatment of penduletin (PDL), an active compound against EV71 we previously reported (data not showed; Zhu et al., 2011). RD cells were infected with EV71 at an MOI of 0.2 for 8 h or 24 h again and then subjected to RT-PCR. The expression of HSPA6 and other three heat shock proteins, including HSPA1A, HSPA8, and HSPB1 and viral genes, were measured. Results showed that EV71 infection significantly upregulated HSPA6 expression in RD cells but had no significant effect on the other three heat shock proteins (Figures 1B,D). The upregulation of HSPA6 in infected cells was completely inhibited by simultaneous treatment of PDL, which was in accord with the expression of viral VP1 gene and positive viral strand (Figures 1B,C). In addition, human brain glioma cells (U251) were normally infected with 1MOI of EV71 (Supplementary Figure S1), and the expression of HSPA6 mRNA in the cells was also found to be upregulated (Figure 1D). These results suggested that EV71 infection specially upregulates the mRNA expression of HSPA6 but not the other three heat shock proteins.

### EV71 Infection Specially Upregulates HSPA6 Protein Expression

**Figure 2:**
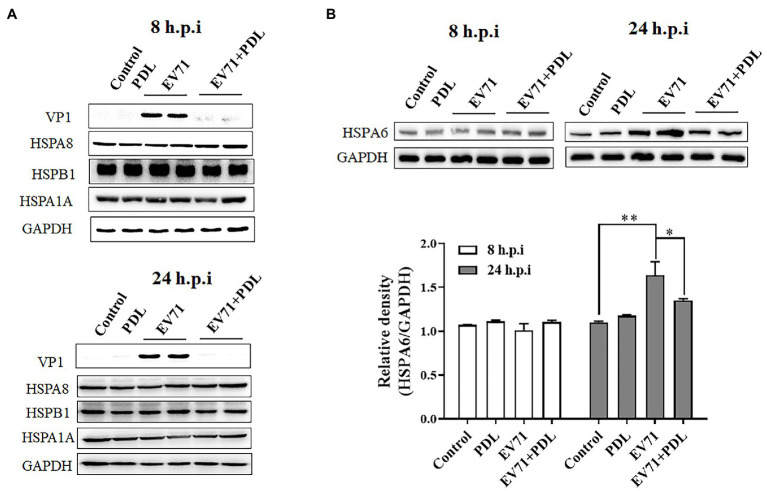
EV71 infection specially upregulates HSPA6 protein expression. **(A)** Western blotting analysis of HSPA1A, HSPA8, HSPB1, and viral VP1 expression in RD cells infected with EV71 for 8 or 24 h. **(B)** Western blotting analysis of HSPA6 expression in RD cells infected with EV71 for 8 or 24 h. The relative expression of HSPA6 was quantitatively analyzed based on the bands’ intensities from the Western blotting analysis. PDL (10 μM) here was used as anti-EV71 control (^*^p < 0.05, ^**^p < 0.01).

To evaluate the inducible effect of EV71 infection on HSPA6 expression at the protein level, RD cells were infected with EV71 for 8 or 24 h and then subjected to Western blotting analysis for HSPA6, HSPA1A, HSPA8, HSPB1, and viral capsid protein VP1. PDL here was used as antiviral control. Results showed that the inducible effect of EV71 infection on HSPA6 and other three heat shock proteins was not significant when detection was performed at 8 h post-infection (hpi; Figure 2). When detection was conducted at 24 hpi, EV71 infection was found to upregulate the expression of HSPA6 significantly, but it did not affect the expression of the other three heat shock proteins (Figures 2A,B). The upregulation of HSPA6 protein caused by EV71 infection was inhibited by the treatment of PDL that possessed strong antiviral activity against EV71. These results are consistent with the observations from the above RT-PCR analysis, which confirms that EV71 infection especially upregulates the expression of HSPA6 at both mRNA and protein levels.

### HSPA6 Knockdown Inhibits EV71 Replication and Protein Expression

**Figure 3:**
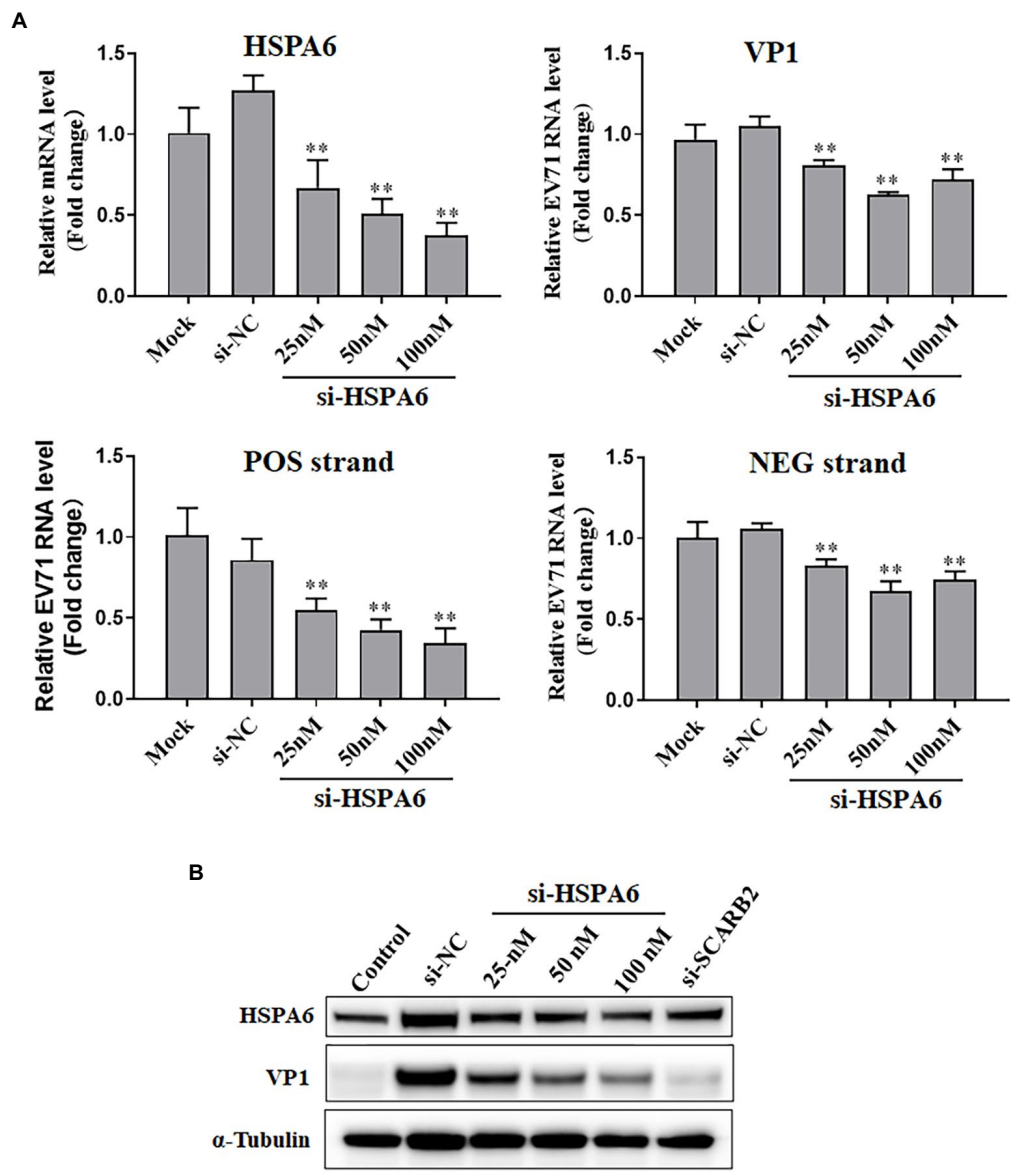
HSPA6 knockdown inhibits EV71 replication and protein expression. **(A)** RT-PCR measurement of HSPA6 as well as viral VP1, positive (POS) strand, and negative (NEG) strand expression in RD cells transfected with siRNAs targeting HSPA6 (si-HSPA6) and infected with EV71. **(B)** Western blotting analysis of protein level of HSPA6 protein and viral VP1 in RD cells transfected with siRNAs and infected with EV71. Mock is the infected but untreated control, si-NC is the negative control siRNA targeting no known genes. Control in B is the cell control without viral infection and siRNA transfection. si-SCARB2 is siRNA targeting the receptor of EV71, using it as the positive control (vs. si-NC, ^**^p < 0.01).

To explore the role of HSPA6 in the infection of EV71, RNAi-directed knockdown of HSPA6 was performed in EV71 infected RD cells, and the viral replication and protein synthesis in the cells were measured. Results showed that siRNA targeting HSPA6 caused a dose-dependent decrease in HSPA6 mRNA level and EV71 VP1 mRNA level and the amount of both viral positive and negative strands (Figure 3A). A similar dose-dependent reduction was also observed in the synthesis of viral protein VP1 (Figure 3B). SCARB2 is an important receptor for EV71 infection. Here siRNA targeting the SCARB2 gene was used as a positive control for RNAi. These results showed that silencing of HSPA6 led to inhibition of EV71 replication and viral protein synthesis, which suggests that HSPA6 plays a role in EV71 infection.

### HSPA6 Knockdown Inhibits EV71 CPE Formation and Daughter Virus Production

**Figure 4:**
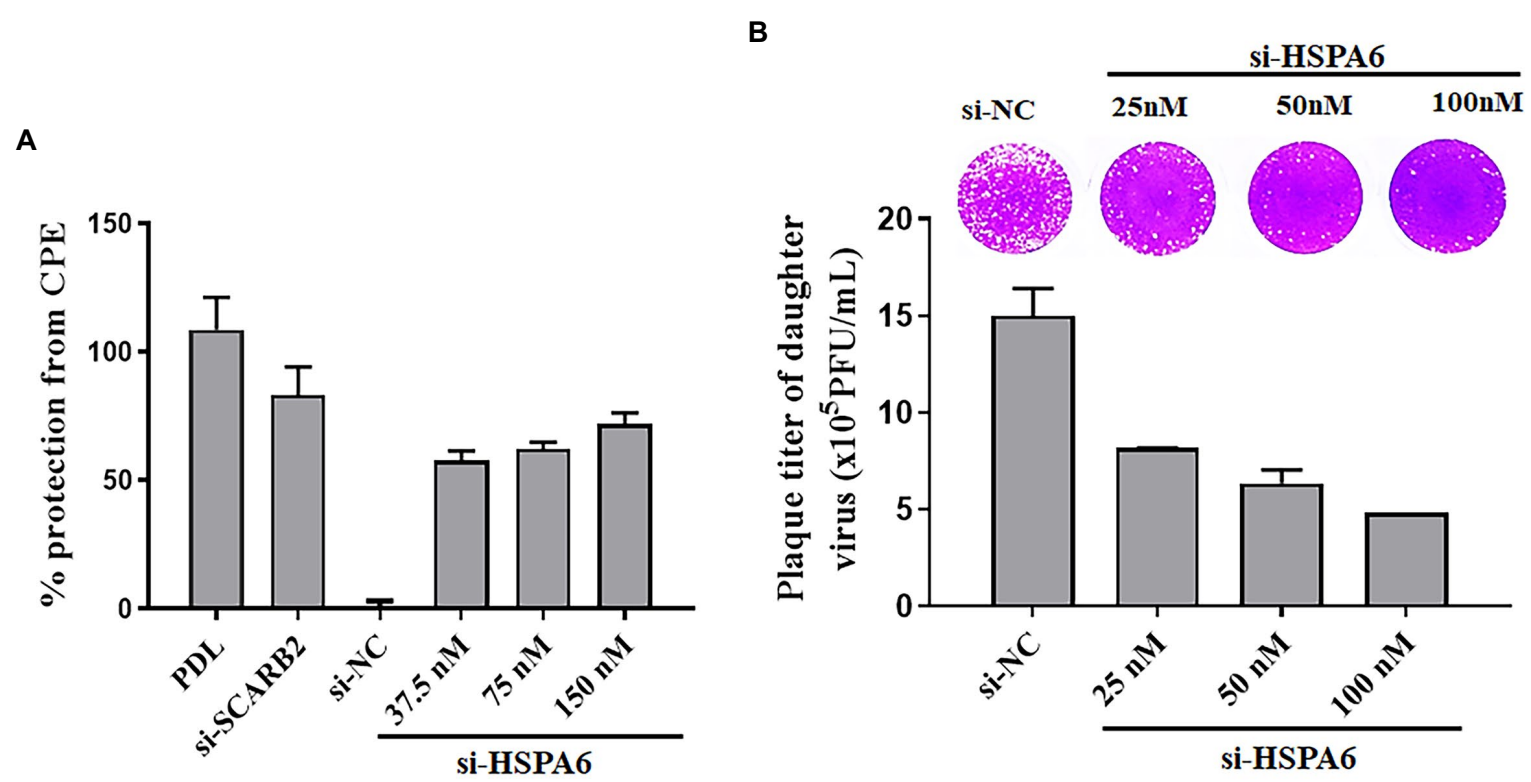
HSPA6 knockdown inhibits EV71 CPE formation and daughter virus production. **(A)** siRNA targeting HSPA6 protected RD cells from EV71-induced CPE. **(B)** siRNA targeting HSPA6 inhibited the production of the EV71 daughter virus. RD cells were transfected with siRNAs for 24 h and then infected with 0.2 MOI EV71 for 72 h, followed by cytopathic analysis. At the same time, the culture supernatant was subjected to plaque reduction assay.

To further study the role of HSPA6 in EV71 infection, the effect of silencing HSPA6 on CPE formation and daughter virus production was investigated. PDL and siRNA targeting SCARB2 were used as positive controls. Results showed that siRNA targeting HSPA6 significantly protected the cells from CPE caused by EV71 infection in a dose-dependent manner (Figure 4A). When the daughter virus production was considered, siRNA targeting HSPA6 significantly reduced the production of the EV71 daughter virus in the culture supernatant (Figure 4B). These results suggest that HSPA 6 plays an important role in EV71 infection.

### EV71 Infection Enhances the Cytoplasmic Aggregation of HSPA6

**Figure 5:**
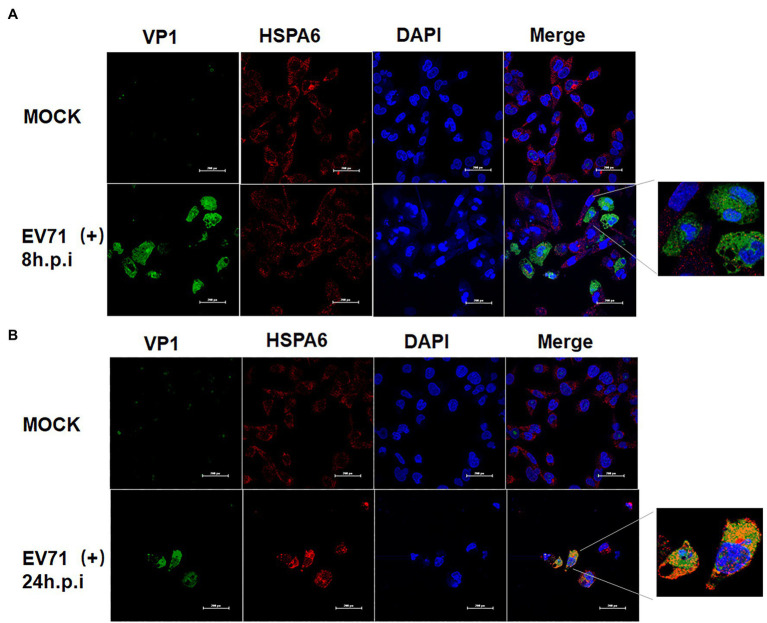
EV71 infection enhances the cytoplasmic aggregation of HSPA6. **(A)** Confocal microscopy analysis of RD cells infected with EV71 at 8 hpi. **(B)** Confocal microscopy analysis of RD cells infected with EV71 at 24 hpi. Cells were stained with VP1/ Alexa Fluor 488, HSPA6/Alexa Fluor 594, and nuclei/DAPI.

To provide a clue for understanding how HSPA6 involve in EV71 infection, the distribution of HSPA6 in EV71 infected cells was investigated with confocal microscopy. RD cells were infected with EV71 for 8 and 24 h, and an immunofluorescence staining was performed with specific antibodies. Results showed that EV71 infection enhanced the cytoplasmic aggregation of HSPA 6 at 24 hpi (Figure 5). Moreover, the aggregated HSPA6 was found to colocalize with viral VP1 protein (Figure 5B). We also observed that HSPA6 cellular levels were significantly elevated at 24 h post-EV71 infection than uninfected cells (Figure 5B). This observation was consistent with the results from RT-PCR and Western blotting performed above.

### HSPA6 Is Involved in the Infection of EV71 but Not Other HFMD-Associated Viruses

**Figure 6:**
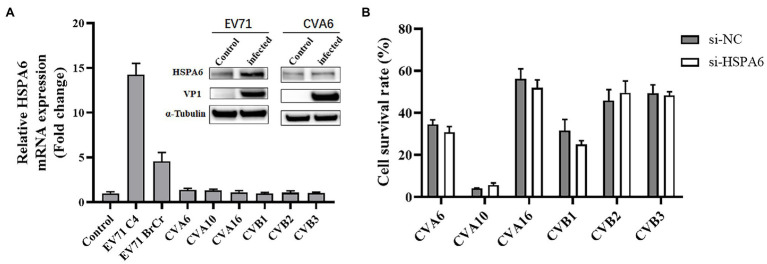
HSPA6 is involved in the infection of EV71 but not other HFMD-associated viruses. **(A)** RT-PCR and Western blotting analysis of HSPA6 expression in RD cells infected with various HFMD viruses. RD cells were infected with 0.2 MOI of EV71 C4 strain, EV71 BrCr strain, CVA6, and CVA10, while Vero cells were infected with 1MOI of CVA16 and CVB1-3. After 8 h infection, the cells were subjected to RT-PCR analysis. Western blotting was also performed to analyze the expression of HSPA6 protein in RD cells infected with 0.2 MOI of EV71 or CVA6 at 36 hpi. **(B)** Survival analysis of RD cells transfected with siRNA targeting HSPA6 and infected with various viruses. RD cells or Vero cells were transfected with 100 nM of si-HSPA6 or si-NC for 24 h and then infected with the viruses for 72 h, followed by the cell survival detection with Alamar blue reagent.

To investigate the inducible effect of other HFMD-associated viruses on HSPA6 expression, RD or Vero cells were infected with different HFMD-associated viruses including EV71 C4 strain, EV71 BrCr strain, CVA6, CVA10, CVA16, and CVB1-3 at MOI of 0.2 for 8 h, and then subjected to RT-PCR analysis for HSPA6 mRNA level. Results showed that only EV71, including strain C4 and BrCr, upregulated the expression of HSPA6, while other viruses showed no obvious effect on HSPA6 expression (Figure 6A). Western blotting was also performed to analyze the expression of HSPA6 protein in RD cells infected with 0.2 MOI of EV71 or CVA6 at 36 hpi. Results showed that the expression of HSPA6 protein was upregulated in RD cells infected with EV71 but not in the cells infected with CVA6. To further investigate the connection between HSPA6 and these non-EV71 HFMD-associated viruses, the cells were transfected with 100 nM of si-HSPA6 or si-NC for 24 h and then infected with the viruses for 72 h, followed by the cell survival detection with Alamar blue reagent. Results showed that silencing of HSPA6 did not affect the infection of CVA6, CVA10, CVA16, and CVB1-3 (Figure 6B), which was different from the observation for EV71 infection. These results suggest that HSPA6 is especially involved in the infection of EV71 but not other HFMD-associated viruses.

## Discussion

HFMD caused by EV71 infection is still a major infectious disease threatening children’s life and health in the absence of effective antiviral drugs due to its high prevalence and severe rate. The study of EV71 infection-related host factors, especially EV71 specific host factors, will help to answer the reasons for the special virulence of EV71 and help find effective ways of antiviral treatment.

PDL is a flavonoid compound with significant anti EV71 activity that we found earlier (Zhu et al., 2011). In the process of tracing the action target of PDL, based on differential transcriptome analysis, we found that EV71 infection can significantly upregulate the expression of HSPA6, including in its most sensitive cell line RD cells and human nervous system-derived U251 cells (Figure 1). Through further research, we found that EV71 only upregulated HSPA6, but not HSPA1, HSPA8, and HSPB1. HSPA1, a major stress-inducible Hsp70 protein member, has been reported to be involved in the infection of Zika virus, Dengue virus, and CVB3 virus (Taguwa et al., 2015, 2019; Wang et al., 2017). HSPA8 is a constitutively expressed Hsp70, the most important Hsp70 protein in Zika virus infection (Taguwa et al., 2019). Moreover, through the knockdown experiment, some studies found that HSPA8 regulates the IRES of EV71 and is required at all stages of the EV71 life cycle (Dong et al., 2018; Su et al., 2020), but here we do not see that EV71 infection can conversely upregulate the expression of HSPA8 (Figures 1, 2). Previously reported that HSPB1 can be upregulated by EV71 infection at a higher MOI and play a supportive role in EV71 replication (Dan et al., 2019). However, we found no significant change in the expression of HSPB1 under a relatively low MOI infection of EV71 (Figures 1, 2). During our study, another group also independently found and first reported the induction effect of EV71 on HSPA6 protein, which is consistent with our findings (Su et al., 2021). They infected RD cells with EV71 C strain and found that HSPA6 is induced to support EV71 replication cycle in an IRES-dependent manner. But the specificity of the induction of HSPA6 was not considered and human nervous system-derived cells were not used in their study. In addition, we further investigate the distribution of HSPA6 in EV71 infected cells and tested the specificity of induction of HSPA6 using other HFMD-associated viruses.

As an important stress response protein of the body, heat shock protein often plays different roles in different viral infections, assists the virus infection, or helps the body resist the virus infection. To further clarify the significance of upregulating HSPA6 in EV71 infection, we downregulated the expression of the HSPA6 gene in cells by RNAi and observed its effect on EV71 virus infection. We found that downregulation of HSPA6 could inhibit the replication of EV71 virus, the synthesis of viral protein, the production of daughter virus, and its cytopathic effect (Figures 3, 4), indicating that HSPA6 is important for the life cycle of EV71 infection. Nevertheless, the mechanism behind it is unclear. A study suggested that HSPA6 may play a role in EV71 infection by promoting the IRES activity of EV71 (Su et al., 2021). Through confocal microscopy analysis, we found that EV71 infection can not only induce HSPA6 expression but also enhance the aggregation of HSPA6 in the cytoplasm and colocalization with viral protein VP1 (Figure 5), suggesting that HSPA6 may be involved in virus assembly.

In addition to EV7, various enteroviruses can cause HFMD in infants, including the most common CVA16, CVA6, and CVA10. To determine whether the induction and dependence of EV71 on HSPA6 are specific in HFMD-associated enteroviruses, we studied the HSPA6 induction effects of six enteroviruses besides two EV71 strains. We found that all of these viruses except EV71 did not significantly upregulate HSPA6 mRNA in RD cells at 8 hpi (Figure 6A). This result is consistent with the detection of HSPA6 at the protein level. At the same time, the expression of HSPA6 was downregulated by RNAi, and there was no significant effect on the cytopathic effect of these viruses except EV71 (Figure 6B). These results suggest that the induction and dependence of EV71 on HSPA6 are specific in these HFMD-associated enteroviruses.

Interestingly, we found that EV71 infection can upregulate the expression of HSPA6 in human neurogliocytes (Figure 1E) but not in neuronal SH-SY5Y cells (data not shown). According to the Human Protein Atlas online database,^1^ HSPA6 is brain-enriched. EV-71 is the most popular and most deadly virus than the other HFDM associated viruses. But the reason why EV71 is more prevalent and more likely to cause severe neurological complications is currently largely unknown. Our findings suggest HSPA6 is a EV71-specific host factor, which hold the potential to be a drug target or biomarkers for EV71 infection. Whether the phenomenon of EV71 specific induction and dependence on HSPA6 is related to the unique neurovirulence of EV71 still needs more in-depth research.

## Conclusion

This study has shown that HSPA6, a member of the host Hsp70 family, was induced by EV71 infection and was involved in EV71 virus replication, protein synthesis, daughter virus production, and cytopathic effect. At the same time, this study, for the first time, found that EV71 has relative specificity in the induction and dependence of HSPA6. Under the same conditions, EV71 did not induce the expression of HSPA1, HSPA8, and HSPB1 genes, and several other common HFMD-associated viruses did not induce the upregulation of HSPA6. This study suggests that HSPA6 is a potential EV71-specific host factor worthy of further study.

## Data Availability Statement

The datasets presented in this study can be found in online repositories. The names of the repository/repositories and accession number(s) can be found at: SRA NCBI, accession number PRJNA803808.

## Author Contributions

JJ planned the experiments and carried out the experiment. GL analyzed the data and wrote the manuscript. JZ and RY carried out the experiment. XS, KZ, and ZR analyzed the data and reviewed the manuscript. ZH supervised the project. QZ conceived the original idea, wrote the manuscript, and acquired financial support. All authors contributed to the article and approved the submitted version.

## Funding

This project was funded by the Science and Technology Program of Guangdong Province (2018A030313252), the Shenzhen Science and Technology Project (JCYJ20170818095732006, JCYJ20190808122605563), and the Shenzhen Peacock Plan and the Fundamental Research Funds for Shenzhen Technology University.

## Conflict of Interest

The authors declare that the research was conducted in the absence of any commercial or financial relationships that could be construed as a potential conflict of interest.

## Publisher’s Note

All claims expressed in this article are solely those of the authors and do not necessarily represent those of their affiliated organizations, or those of the publisher, the editors and the reviewers. Any product that may be evaluated in this article, or claim that may be made by its manufacturer, is not guaranteed or endorsed by the publisher.

## Footnotes

^1^ www.proteinatlas.org
