# Supplementary material for: Heat Shock Protein A6 Is Especially Involved in Enterovirus 71 Infection
Source: Front Microbiol. 2022 Mar 4;13:865644. doi: 10.3389/fmicb.2022.865644 (PMC8931677; doi:10.3389/fmicb.2022.865644)
Supplement: Supplementary file 1 [file Data_Sheet_1.DOCX]

Supplementary Material

# Supplementary Figures


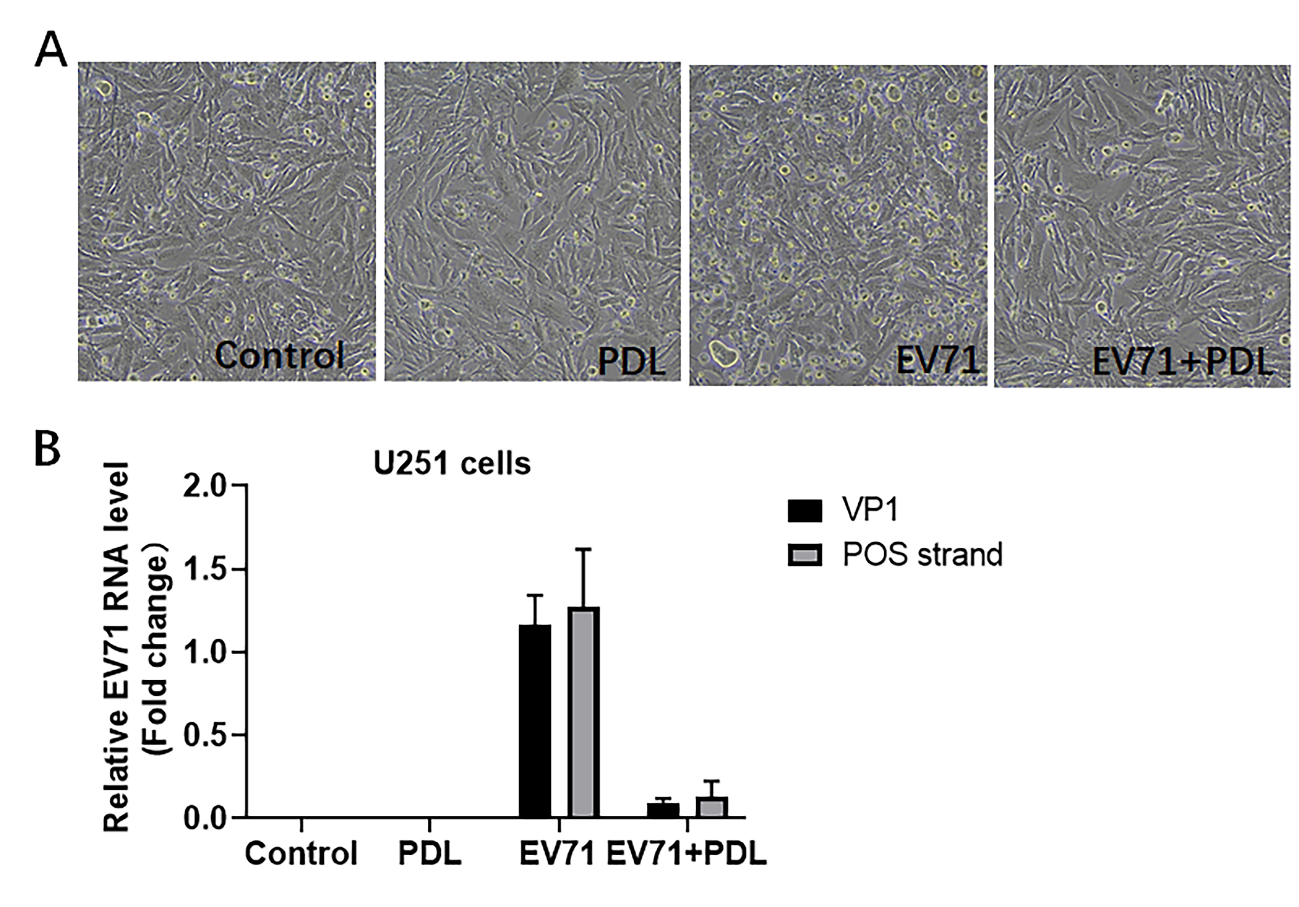


**Supplementary Figure 1.** **U251 cells were infected with EV71.** (A) Cytopathic effect in U251 cells infected and/or treated with PDL for 48 h. (B) RT-PCR measurement of viral VP1 and positive-strand in U251 cells infected and/or treated with PDL for 24 h. U251 cells were infected with EV71 at an MOI of 1 and/or treated with 10μM of PDL. After 24 h or 48 h, the cells were subjected to RT-PCR or microscopy observation.
